# Healthcare workers’ attitudes and factors affecting provision of sexual and reproductive health services to adolescents in refugee settlements, Western Uganda

**DOI:** 10.3389/frph.2025.1498305

**Published:** 2025-05-08

**Authors:** Simon Binezero Mambo, Moazzam Mohiuddin Lodhi, Isa Asiimwe, Gloria Neema Bizimana, Amos M’yisa Makelele, Solomom Adomi Mbina, Mathew Chibunna Igwe, Umar Ibrahim

**Affiliations:** ^1^ Department of Public Health, School of Allied Sciences, Kampala International University Western Campus, Ishaka Bushenyi, Uganda; ^2^ Youth Alliance for Reproductive Health, Goma, Democratic Republic of Congo; ^3^ Department of Paediatric, Faculty of Clinical Medicine and Dentistry Kampala International University Western Campus, Ishaka, Bushenyi, Uganda; ^4^ Department of Medical Laboratory Science, Faculty of Allied Health Sciences, Enugu State University of Science and Technology, Enugu, Nigeria

**Keywords:** healthcare workers, sexual and reproductive health services, adolescents, refugee settlements, western Uganda

## Abstract

**Background:**

Provision of sexual and reproductive health services has an important impact on the adolescents standard of life. Healthcare workers interactions with clients has been highlighted as one of the main barriers keeping adolescents from seeking sexual and reproductive health services. This study aimed to assess the Healthcare workers’ attitudes and factors affecting provision of sexual and reproductive health services in the Nakivale, Kyaka II, and Rwamwanja refugee settlements.

**Methods:**

This was a cross-sectional quantitative study in which healthcare workers from public and private health facilities refugee communities in south-western Uganda responded to a questionnaire. Binary logistic regression was done to assess the baseline characteristics associated with provision of these services using SPSS version 26.

**Results:**

Of the 386 medical professionals enrolled, 194(50.3%) were females with a mean age of 30.9 years (SD = 6.9). The services that were most commonly offered were contraception counselling/provision (81.3%) and comprehensive sexuality education provision (75.1%). The least offered services were safe abortion care (40.9%). The good attitudes were highest towards comprehensive sexuality education and antenatal, intrapartum plus postnatal care while the bad attitudes were highest toward safe abortion care. Residence, designation and type of facility had a significant association with offering of the different services (*P* < 0.05 for all at multivariate level of analysis). Being from rural area was positively associated with ASRH services (cOR = 2.685, 95% CI = 1.414–5.098). Being a nurse was associated with reduced provision of services as compared to being a counselor (cOR = 0.295, 95% CI = 099–0.882). Government facilities were more likely to offer adolescent sexual and reproductive health services compared to private facilities (cOR = 2.155, 95% CI = 1.169–4.075).

**Conclusion:**

In this study, majority of the study participants had a good attitude towards comprehensive sexuality education provision and antenatal, intrapartum and postnatal care while the bad attitudes were highest toward safe abortion care. More efforts are still required toward provision of safe abortion care and harmful traditional practices prevention. This will be achieved by providing training which will be vital in improving knowledge and attitude toward these services. The training should be more focused on the older professionals and non-councilors.

## Introduction

Globally there are about 1.3 billion of adolescents, between the ages of 10 and 19 years, nine out of 10 people in this age-group live in less developed countries (1, 2). Adolescent sexual and reproductive health (ASRH) care needs are rising in both low and middle-income countries, especially for young women and marginalized groups such as refugees, faces unique challenges, including long waiting times at health facilities, lack of privacy, mistreatment and general poor quality of services (3). Such factors deter these patients from using health services, ultimately placing them at risk for negative SRH outcomes, including HIV infection, unintended pregnancy and unsafe abortion (4). In Uganda, more than 30% of the total population is aged 10–24 years, making it one of the youngest countries in the world, with 1,252,470 refugees and asylum seekers, Uganda is the third refugee-hosting nation in the world after Ethiopia and Kenya (5). Uganda's approach to refugee management is recognized as one of the most progressive in the world. The Office of the Prime Minister (OPM), specifically the Department of Refugees, plays a central role in refugee management, have put in place a system that ensures refugees living in settlements have access to healthcare services and facilities per Ministry of Health guidelines (5, 6).

Humanitarian settings in Uganda are associated with high prevalence of gender based violence (GBV) which is a risk factor for poor ASRH outcomes (7). In Uganda, ASRH services such as HIV and AIDS, and contraceptive use are often neglected, moreover, access and availability to ASRH is limited due to social and structural factors and over-bur dened health systems within humanitarian settlements lack of respect for adolescents' privacy and confidentiality, and the ill treatment by healthcare workers are some of the reported negative behaviors that discouraged sexually active adolescents from seeking SRH services (7, 8). Healthcare workers interactions with clients has been highlighted as one of the main barriers keeping adolescents from seeking SRH services (8). In humanitarian settings, international recommendations emphasize giving priority to the provision of SRH information and services (9). Adolescent girls continue to have poor understanding of and access to SRH services in humanitarian situations like the Nakivale settlement (3–10).

Provision of sexual and reproductive health services has an important impact on the adolescents standard of life (11). These services must be available, and staff qualities like competence, secrecy, and provider privacy, as well as attitudes, play a significant role in how often people use them (8–12). Settlements of refugees are highly susceptible to problems with sexual and reproductive health (13). The study give insight about health care workers' attitudes toward adolescent sexual and reproductive health in humanitarian settings could be helpful in designing appropriate intervention measures to improve adolescent sexual and reproductive health in the settlements.

## Methods

### Study design and setting

We conducted a cross-sectional survey in 3 refugee settlements of the south-western Uganda, specifically Nakivale, Kyaka II and Rwamwanja. All 3 settlements had one health Centre IV government-led facility and privately owned facilities. Healthcare workers of the different cadres, Doctors, Clinical officers, nurses, midwives and counselors both from Public and private facilities were enrolled into the study.

### Sample size and sampling

Kish and Leslie formula was used to identify the necessary minimum sample size. *n* = z^2^(pq)/e^2^. Since no quantitative study had been done in Uganda on the subject, 50% was used for the proportion. Therefore *P* = 0.5, q = 0.5, e = 0.05 and z = 1.96. On substituting, *n* = 385.1 = 386. Therefore, a minimum of 386 participants was required.

All health workers that agreed to participate in the research were enrolled after signing an informed consent document until the sample size was attained.

### Data collection procedure

Data were collected using a self-administered structured questionnaire provided to respondents at their respective health institutions. The questionnaire was adapted from Tilahun et al. (13) and pretested on a convenience sample of 20 health workers that were not included in the study and corrections were made afterwards. The sociodemographic characteristics of the healthcare provider (age, sex, tribe, religion, place of residence, level of education, and marital status) as well as the attitude of the healthcare provider (positive (supportive, understanding) or negative (judgmental, stigmatizing, abusive, stereotyping) were the independent variables. Factors connected to health facilities (availability of services and availability of staff) were the intervening variables. Provision of the SRH services was the dependent variable.

The participant were consecutively selected in the health facilities for the 3 settlements, they gave information on the sexual and reproductive health services offered to the adolescents. The participants also responded to the questions relating to their attitude toward provision of each of the services. The questions relating to factors affecting provision of sexual and reproductive health services were also responded to. Data collection took place from August to October, 2023.

### Statistical analysis

Information was summarized in excel and entered into SPSS version 26 for analysis. The proportion of health workers providing specific services was computed. The proportion of health workers with a specific attitude category was computed. The proportion of factors reported by health workers to affect provision of sexual and reproductive services to adolescents was computed. To measures attitude and Factors affecting provision of services, Compute descriptive statistics were done with the Percentage of participants who checked response the to facilitate interpretation and comparison. Bivariate analysis was done for the study participants' characteristics against provision of specific adolescent sexual and reproductive health care services. Variables with *p*-value below 0.2 at bivariate were re analyzed at multivariate using binary logistic regression to determine health worker related factors significantly affecting provision of sexual and reproductive health care services. *P*-value less or equal to 0.05 was regarded as significant.

### Ethical clearance

The Instituttional Research Ethics Review Committee of Bishop Stuart University approved the protocol with the number BSU REC 2023–203 and authorization to collect data from Refugee settlements was granted by Office of the Prime Minister refugee department were granted with the reference No OPM/41/1. The healthcare workers were provided information about the study and its importance, and confidentiality of the information requested. Written consent was then obtained. from participants in a form provided with the study questionnaire.

## Results

### Baseline characteristics of the study participants

In this study that enrolled 386 medical professionals, majority were females 194(50.3%) with a mean age of 30.9 years (SD = 6.9), from rural areas 335(86.8%). Majority were nurses 227(58.8%), certificate holders 183(47.4%) from private health facilities 279(72.3%).

**Figure 1:**
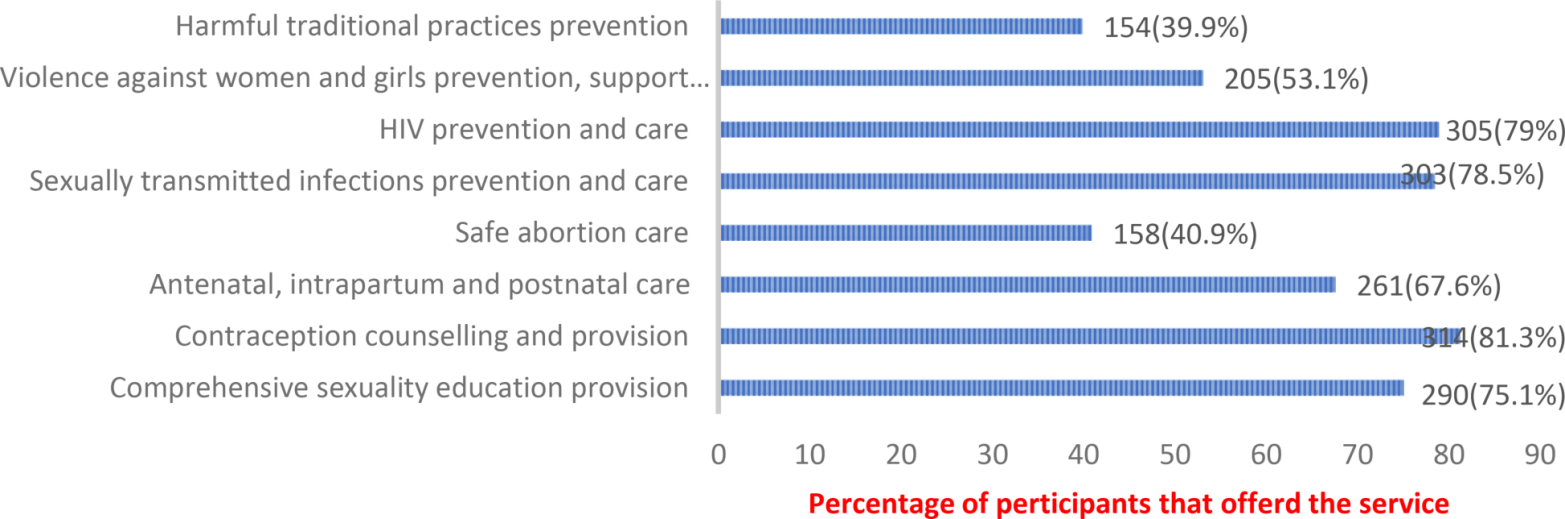
Adolescent sexual and reproductive health services offered by healthcare professionals in the refugee camps of Nakivale, Kyaka II, and Rwamwanja.

The services provided are shown in Figure 1. The service that was most commonly offered was contraception counselling and provision offered by 81.3% of the participants, followed by HIV prevention and care (79.0%), sexually transmitted infections prevention and care (78.5%) and comprehensive sexuality education provision (75.1%). The least offered services were safe abortion care offered by only 40.9% of the study participants and harmful traditional practices prevention offered by only 39.9% of the participants.

Table 1: provides information about the majority of the study participants had a good attitude regarding offering services for adolescent sexual and reproductive health as evidenced by their responses on the questions regarding attitude. Majority responded that “*adolescents need to be helped*” and “*adolescents need to be educated*”. The good attitudes were highest towards comprehensive sexuality education provision and antenatal, intrapartum and postnatal care with about 80% of the participants reporting that adolescents that need these services need to be helped and educated. The bad attitudes were highest toward safe abortion care with 21.8% of the participants reporting that “*they don*’*t want to help adolescents who need safe abortion care*”.

**Table 1:** Attitudes of healthcare workers regarding offering services for adolescent sexual and reproductive health in the refugee camps of Nakivale, Kyaka II, and Rwamwanja.

ASRH services	Comprehensive sexuality education provision	Contraception counselling & provision	Antenatal, intrapartum and postnatal care	Safe abortion care	STI prevention and care	HIV prevention and care	Violence against females prevention and care	Harmful traditional practices prevention
Attitude	Percentage of participants who checked response							
They are careless	19.4	19.9	17.4	**29**.**5**	23.8	19.9	16.3	14.0
They have no morals	21.8	22.3	17.4	**31**.**6**	20.7	17.6	14.8	17.1
They were poorly brought up	29.5	23.8	22.0	**26**.**4**	18.1	16.6	19.7	19.7
They will be punished by God	18.7	16.8	13.5	**27**.**2**	16.1	13.5	12.4	13.2
They are irresponsible	22.8	20.7	13.7	**25**.**6**	21.0	14.8	13.0	14.5
They are stupid	14.2	12.7	12.2	**16**.**8**	12.2	14.0	13.0	13.0
They need to be helped	**79**.**0**	**71**.**2**	**80**.**8**	**60**.**9**	**76**.**9**	**75**.**6**	**76**.**4**	**74**.**1**
They need to be educated	**80**.**8**	**79**.**3**	**79**.**8**	**70**.**5**	**80**.**3**	**82**.**4**	**81**.**6**	**74**.**6**
I don't want to help them	13.2	15.5	14.0	**21**.**8**	14.0	16.1	16.1	13.7
Mistakes are part of Life	21.0	19.2	15.5	**22**.**0**	21.5	16.6	20.2	16.6
Others	1.6	2.3	1.8	**1**.**8**	2.6	1.8	1.0	1.0

The bold value shows factors which affect service provision by having highest percentage of participants.

Table 2: Provides information on the main factor that hindered delivery of adolescent sexual and reproductive health services that cut across all services was inadequate training with up to 62.7% of the participants reporting that they had inadequate training regarding harmful traditional practices prevention. Compared to other services, safe abortion care was more hindered by contradiction with beliefs of medical professionals reported by 32.6% of the study participants, contradiction with culture of medical professionals (31.6%), contradiction with faith/religion of medical professionals (43.8%) and fear of imprisonment (19.4%). Compared to other services, violence against women and girls prevention was more hindered by “other” factors which were not explored in this study (41.7%).

**Table 2:** Factors impacting the delivery of adolescent sexual and reproductive health services in the Nakivale, Kyaka II, and Rwamwanja refugee camps.

ASRH services	Comprehensive sexuality education provision	Contraception counselling & provision	Antenatal, intrapartum and postnatal care	Safe abortion care	STI prevention and care	HIV prevention and care	Violence against females prevention and care	Harmful traditional practices prevention
Factor/reason	Percentage of participants who checked response							
Inadequate training	**55**.**4**	**41**.**2**	**42**.**7**	**40**.**7**	**42**.**0**	**39**.**9**	**41**.**7**	**62**.**7**
Contradiction with my values	26.9	24.6	18.4	28.5	18.4	15.8	21.8	22.0
Contradiction with my beliefs	25.6	24.4	18.7	**32**.**6**	19.9	18.4	16.3	23.3
Contradiction with my culture	22.3	25.9	20.7	**31**.**6**	18.9	17.4	20.2	23.3
Contradiction with faith/religion	25.6	32.6	18.1	**43**.**8**	17.9	23.1	22.8	25.1
Services are not available	24.6	30.1	21.5	31.6	18.9	26.4	26.7	31.3
Services are expensive	26.7	25.9	37.0	28.0	28.2	27.7	23.6	21.2
No time to offer the services	17.4	11.1	8.3	8.0	7.3	9.3	16.1	15.3
I will be imprisoned	2.8	1.3	7.3	**19**.**4**	2.6	3.6	5.2	4.1
Others	2.8	5.4	4.4	3.1	5.7	7.0	**41**.**7**	4.4

The bold value shows factors which affect service provision by having highest percentage of participants.

Table 3: Both bivariate and multivariable analyses were conducted regarding the association between baseline characteristics and adolescent sexual and reproductive health services.The study showed that residence, designation of medical professional and type of health facility had a statistically significant association with offering of the different adolescent SRH services (*P* < 0.05 at multivariate level of analysis). Being from rural area was positely associated with ASRH services (cOR = 2.685, 95% CI = 1.414–5.098). Being a nurse was associated with reduced provision of services as compared to being a counselor (cOR = 0.295, 95% CI = 099–0.882). Government facilities were more likely to offer adolescent sexual and reproductive health services compared to private facilities (cOR = 2.155, 95% CI = 1.169–4.075).

**Table 3:** Association between baseline characteristics and sexual and reproductive health services provision.

Characteristic	Bivariate analysis			Multivariate analysis		
	cOR	95% CI	*P*-value	aOR	95% CI	*P*-value
Age						
19–30	1.008	0.974–1.043	0.636			
Above 30years	Ref.					
Sex						
Male	Ref					
Female	1.072	0.675–1.700	0.769			
Residence						
Rural	**2**.**676**	**1.452–4.931**	**0**.**002**	**2**.**685**	**1.414–5.098**	**0**.**003**
Urban	Ref					
Education						
Primary	0.452	0.036–5.707	0.539			
Secondary	0.516	0.154–1.729	0.284			
Certificate	0.653	0.270–1.583	0.346			
Diploma	0.697	0.282–1.727	0.436			
Degree	Ref					
Designation						
Counselor	Ref					
Nurse	**0**.**300**	**0.102–0.878**	**0**.**028**	**0**.**295**	**0.099–0.882**	**0**.**029**
Clinical officer	0.329	0.104–1.046	0.060	0.338	0.104–1.102	0.072
Lab technician	0.412	0.090–1.881	0.252	0.371	0.080–1.729	0.207
Doctor	1.882	0.194–18.228	0.585	1.564	0.159–15.394	0.701
Other	0.275	0.050–1.508	0.137	0.156	0.026–0.922	0.040
Religion						
Anglican	0.689	0.372–1.276	0.236			
Catholic	0.793	0.429–1.468	0.461			
Muslim	3.080	0.669–14.171	0.249			
Other	Ref					
Type of facility						
Government	**2**.**183**	**1.169–4.075**	**<0**.**014**	**2**.**155**	**1.152–4.031**	**0**.**016**
Private	Ref					

aOR, adjusted odds ratio; Ref, reference category, CI, confidence interval.

The bold value shows factors which affect service provision by having highest percentage of participants.

## Discussion

This study assessed the healthcare workers attitudes and practices in addition to the factors affecting adolescent sexual and reproductive health care in the Nakivale, Kyaka II, and Rwamwanja refugee settlements. The study enrolled 386 medical professionals, majority were nurses, from private health facilities. We noted that the service that was most commonly offered was contraception counselling and provision, followed by HIV prevention and care, sexually transmitted infections prevention and comprehensive sexuality education provision. The least offered services were safe abortion care and harmful traditional practices prevention. In agreement with our findings, a systematic analysis of the data on adolescent sexual and reproductive health services discovered that the majority of services offered in refugee populations focused on HIV/STIs and maternal and newborn health (14). The review found that services like prevention of Harmful traditional practices, provision of safe abortion and post-abortion care were not commonly offered (14). The services like contraception counselling/provision, HIV prevention/care, sexually transmitted infections prevention and comprehensive sexuality education provision were part of the care packages for all patients irrespective of the age which can explain why they were the most commonly offered. The possible reason for low safe abortion care is the controversy that surrounds abortion in terms of the country laws as well as the beliefs and norms of the different communities. The low harmful traditional practices prevention services female genital mutilation are most likely as a result of inadequate knowledge and cultural practices in refugees community.

Contrally to our findings, a systematic analysis of the data on adolescent sexual and reproductive health services discovered that the majority of services offered in refugee populations focused on gender-based violence (14, 15), yet in our study only about 53% of the respondents offered services relating to violence against women and girls. This low provision of such services could be because of low knowledge however, about 41% of the participants reported that there were other challenges which affected these services that were not explored in this study.

In this study, majority of the study participants had a good attitude regarding offering services for adolescent sexual and reproductive health. The good attitudes were highest towards comprehensive sexuality education provision and antenatal, intrapartum and postnatal care while the bad attitudes were highest toward safe abortion care. The fact that antenatal, intrapartum and postnatal care is part of routine care offered to all patients could have contributed to the good attitudes. In agreement with our findings, in a South African study, nurses said they had trouble understanding and implementing the notion of abortion (16). They clarified that their traditional ideas and values were in conflict with this practice, which resulted in a negative attitude toward the adolescents who came in need of this assistance (16).Non-marital sex and its repercussions, such as pregnancy, abortion, and sexually transmitted illnesses, were noted to be viewed as immoral, disrespectful, and disobedient in Ghana (17), which is similar to many cultures in Uganda and across Africa.

We also observed that the main factor that hindered delivery of adolescent sexual and reproductive health services that cut across was inadequate training. In agreement with our findings, lack of knowledge of SRH, was one of the main obstacles preventing the use of SRH in Ethiopia (18). Compared to other services, safe abortion care was more hindered by contradiction with beliefs of medical professionals, contradiction with culture, contradiction with faith/religion and fear of imprisonment. In agreement with this, negative cultural attitudes was one of the main barriers blocking young people in the Lao People's Democratic Republic from utilizing sexual and reproductive health services (3). Also a thorough investigation focusing on Africa reported that one of the main limiting factors was stigma (19).

Increasing age of the medical professional was associated with less contraception counselling and provision which was in agreement with a study done in Nigeria where access to sexual and reproductive health care was shown to be substantially correlated with age (20). This may be because older people are more likely to have stronger beliefs in culture and tradition as compared to younger people.

Rural residence was associated with increased provision of majority of the services. This was in agreement with findings by (18) in Ethiopia who reported that area of residence was substantially linked with the application of sexual and reproductive health services. This could be because in urban areas, patients are more likely to seek care from private facilities, which brings in the issue of costs as was seen in Congo, where Urban participants brought up costs as the hindrance to these services (21)

Being a nurse or doctor was associated with reduced provision of services as compared to being a counselor, Doctors and nurses focus on physical health: diagnosing and treating illnesses, providing vaccinations, managing injuries, and ensuring basic medical care while Counselors their strength lies in providing psychosocial support, education, addressing trauma and other psychological needs for adolescents (22). Also lower education level was associated with less contraception counselling and provision. In Nigeria access to sexual and reproductive health care was shown to be substantially correlated with education (23) This association could be arising as a result of knowledge about the subject. Counselors were more likely to offer these services possibly because they learn to understand every person and to avoid being judgmental which could have improved their attitude towards provision of these services.

Government facilities were more likely to offer adolescent sexual and reproductive health services compared to private facilities. A thorough investigation focusing on Africa reported that one of the main limiting factor was cost (3). Since government facilities offer these services for free, it is understandable that they were more likely to offer these services. Also private facilities are aimed at maximizing profit which could explain why they would provide less of these services given that most of the adolescents may not have the funds to cater for these services even when they need them. This could be because they fear to get money from their parents or guardians for such services in fear of judgment or punishment.

### Strength and limitations

This was the first multicentre study in Uganda that assessed the healthcare workers' attitudes and factors affecting provision of sexual and reproductive health services to adolescents in refugee settlements of western Uganda.

This was a cross sectional study in which conclusions about causation could not be made. Compared to other services, violence against women and girls prevention was more hindered by “other” factors which were not explored in this study.

## Conclusion

In this study, majority of the study participants had a good attitude regarding offering services for adolescent sexual and reproductive health. The good attitudes were highest towards comprehensive sexuality education provision and antenatal, intrapartum and postnatal care while the bad attitudes were highest toward safe abortion care. The services that was most commonly offered were contraception counselling and provision, HIV prevention and care, sexually transmitted infections prevention and comprehensive sexuality education provision. The least offered services were safe abortion care and harmful traditional practices prevention. The main factor that hindered delivery of adolescent sexual and reproductive health services that cut across all services was inadequate training. Residence, designation of medical professional and type of health facility had a statistically significant association with offering of the different adolescent sexual and reproductive health services. More efforts are still required toward provision of safe abortion care and harmful traditional practices prevention.

## Data Availability

The raw data supporting the conclusions of this article will be made available by the authors, without undue reservation.
